# The histone demethylase enzyme KDM3A is a key estrogen receptor regulator in breast cancer

**DOI:** 10.1093/nar/gku1298

**Published:** 2014-12-08

**Authors:** Mark A. Wade, Dominic Jones, Laura Wilson, Jacqueline Stockley, Kelly Coffey, Craig N. Robson, Luke Gaughan

**Affiliations:** 1 Northen Institute for Cancer Research, Newcastle University, Newcastle upon Tyne NE2 4HH, UK; 2 The Beatson Institute for Cancer Research, Garscube Estate, Switchback Road, Bearsden, Glasgow G61 1BD, UK

## Abstract

Endocrine therapy has successfully been used to treat estrogen receptor (ER)-positive breast cancer, but this invariably fails with cancers becoming refractory to treatment. Emerging evidence has suggested that fluctuations in ER co-regulatory protein expression may facilitate resistance to therapy and be involved in breast cancer progression. To date, a small number of enzymes that control methylation status of histones have been identified as co-regulators of ER signalling. We have identified the histone H3 lysine 9 mono- and di-methyl demethylase enzyme KDM3A as a positive regulator of ER activity. Here, we demonstrate that depletion of KDM3A by RNAi abrogates the recruitment of the ER to *cis*-regulatory elements within target gene promoters, thereby inhibiting estrogen-induced gene expression changes. Global gene expression analysis of KDM3A-depleted cells identified gene clusters associated with cell growth. Consistent with this, we show that knockdown of KDM3A reduces ER-positive cell proliferation and demonstrate that KDM3A is required for growth in a model of endocrine therapy-resistant disease. Crucially, we show that KDM3A catalytic activity is required for both ER-target gene expression and cell growth, demonstrating that developing compounds which target demethylase enzymatic activity may be efficacious in treating both ER-positive and endocrine therapy-resistant disease.

## INTRODUCTION

Approximately two-thirds of newly diagnosed breast cancers (BCa) express estrogen receptor-α (ERα, hereafter called ER) and require ER-mediated transcriptional activation for tumour growth. Therapy for ER-positive BCa has focussed on abrogating ER activity by preventing binding of the ER to its activating hormone estrogen (1). Unfortunately, cancers become resistant to such endocrine therapies and progress due to poorly defined molecular events that enable ER function in the absence of ligand (2). Evidence suggests that fluctuations in the activity of ER co-regulatory proteins play a role in BCa progression and could facilitate resistance to therapy (3–7). Developing therapies which target ER co-regulators may therefore provide effective ways of treating ER-positive BCa.

Histone lysine methylation is an important regulator of transcription and aberrant methylation patterns have been associated with oncogenesis (8,9). Mono-/di-/tri-methylation (me1/2/3) of specific lysines in histones H3 and H4 play an important role in regulating gene expression by altering chromatin structure to activate or repress transcription (10,11). Histone methyltransferases (HMTs) are a family of SET domain-containing enzymes that catalyse the addition of methyl groups to distinct lysine residues on histones H3 and H4. Removal of histone methylation is catalysed by histone demethylase (HDM) enzymes (12,13). There are eight characterized HDM enzyme families (termed KDMs) all of which, with the exclusion of KDM1, contain a Jumanji-C (JmjC) demethylase domain (14). Both HMT enzymes and HDM enzymes have been directly associated with ER regulation and BCa development. For example, the HMT EZH1 is overexpressed in BCa, the HDM KDM4C promotes BCa cell growth and metastasis, and the HDMs KDM1 and KDM4B are both required for ER-mediated transcription (15–21). KDM4B is also required for BCa cell growth and expression of the ER and ER pioneer proteins (19,20,22). These findings suggest a role for dysregulated histone methylation in BCa development and identify HMTs and HDMs as potential therapeutic targets.

Using an siRNA screen we identified that the HDM KDM3A was required for ER target gene expression. KDM3A is a member of the 2-oxyglutarate/Fe(II)-dependent JmjC family of HDMs that demethylate transcriptionally repressive H3K9 mono- and di-methyl marks (23,24). KDM3A is up-regulated by HIF-1α during hypoxia and KDM3A expression is elevated in both bladder and lung cancer (25–27). Depletion of KDM3A has been shown to reduce bladder, lung, colon and hepatocellular carcinoma cell growth (25,27,28). KDM3A also regulates androgen receptor (AR) activity in prostate cancer cell lines (23).

Here, we show that KDM3A depletion reduces ER-target gene expression and abrogates the recruitment of the ER to *cis*-regulatory elements within target gene promoters. Microarray analysis determined that genes down-regulated by KDM3A depletion play crucial roles in cell growth and this was supported by proliferation assays in BCa cell lines. Importantly, the catalytic activity of KDM3A is crucial for both ER-target gene expression and cell proliferation in breast cancer. Furthermore, we have demonstrated that KDM3A knockdown inhibits ER-target gene expression and cell proliferation in a model of endocrine therapy-resistant BCa. Together, our findings identify that KDM3A is essential for ER signalling and confirm KDM3A as an important BCa therapeutic target.

## MATERIALS AND METHODS

### Cell culture

MCF-7, T47D, BT-474, ZR751 and HEK293 cells were maintained in RPMI-1640 media (Sigma) containing 10% foetal-calf serum (FCS) (Gibco) and 1% penicillin/streptomycin (Sigma). For estrogen stimulation assays, cells were grown in phenol red-free RPMI-1640 media (Gibco) supplemented with 10% serum stripped FCS (Hyclone) and 1% penicillin/streptomycin for 24 (KDM3A knockdown chromatin immunoprecipitation (ChIP) experiments) or 48 h (ChIP/gene expression/microarray experiments) prior to the addition of 10 nM 17-β-estradiol (E_2_) (Sigma) for 45 min (ChIP) or 4 h (gene expression/microarray analysis). MMU2 cells were maintained in phenol red-free RPMI-1640 media supplemented with 10% dialysed serum (Gibco) and 1% penicillin/streptomycin. MCF-10A cells were maintained in DMEM-F12 (Sigma) media containing 5% horse serum (Sigma), 10 μg/ml insulin (Sigma), 0.5 μg/ml EGF (Sigma), 100 ng/ml cholera toxin (Sigma) and 1% penicillin/streptomycin.

### siRNA transfection

The initial siRNA library screen was conducted as described in (19). Two KDM3A targeting siRNAs (siKDM3A-B and siKDM3A-C), an ER targeting siRNA (siER) and a non-silencing scrambled control siRNA (siSCR) were utilized in this study. All siRNAs were purchased from Sigma and sequences are shown in Supplementary Table S2. Cell lines were transfected with individual siRNAs using Lipofectamine RNAiMAX (Invitrogen) to a final concentration of 25 nM according to manufacturer's instructions and as previously described (29). To assess KDM3A and ER expression and global H3K9me1/2 status, protein from transfected cells was harvested in SDS-sample buffer and subject to polyacrylamide gel electrophoresis (SDS-PAGE) prior to immunoblotting with specific antibodies as described in (30) (antibody details: Supplementary Table S3). For gene expression analysis, RNA was extracted using TRIzol (Ambion, Life Technologies) and cDNA was generated to be analysed by quantitative PCR (qPCR) as previously described (29) (for primer sequences see Supplementary Table S4).

### Microarray analysis

Transfections were set up and RNA extraction performed as described above. Gene expression data were obtained by hybridizing triplicate samples to Illumina HT-12 version 4 BeadChips, although one sample failed due to low amount of cRNA (siKDM3A + E_2_). Raw data for the remaining 11 samples were processed and analysed using R statistical software (http://www.R-project.org) and BioConductor (31) packages as described below. Background corrected signal intensities were variance-stabilized and normalized using the ‘vsn’ package (32). Quality control plots did not reveal any further outlier samples. The dataset was then filtered to remove probes not detected (detection score <0.95) in any of the samples, resulting in a final dataset of 28 909 probes.

Statistical analysis was performed using the Linear Models for Microarray Analysis (limma) package (33). Comparisons to find genes changing in response to estrogen stimulation in the control and knockdown groups, as well as differences between the control and knockdown groups, were performed. Multiple testing correction was applied using the false discovery rate controlling procedure of Benjamini and Hochberg (34). Probes with adjusted *P*-values <0.05 were considered significantly differentially expressed and results were annotated with the annotation package illuminaHumanv4 (Dunning M, Lynch A and Eldridge M. *illuminaHumanv4.db: Illumina HumanHT12v4 annotation data (chip illuminaHumanv4*). R package version 1.22.1).

### Gene ontology analysis

Gene lists were analysed using the Functional Annotation tool of the DAVID Bioinformatics Resource v6.7 (35,36) and against a background of the Illumina HumanHT-12 gene list. Analysis was performed using default settings and the SP_PIR_KEYWORDS, GOTERM_BP_FAT (Biological Processes), GOTERM_MF_FAT (Molecular Functions) and KEGG_PATHWAY databases. The top 20 enriched gene ontology (GO) terms ranked by their Fisher Exact *P*-values are shown in Supplementary Table S1. Fold enrichment indicates the number genes found in the gene list for each GO term compared to the number you would expect by random chance from a gene list of the size analysed.

### Chromatin immunoprecipitation

Chromatin was harvested from MCF-7 and T47D cells and fractionated by sonication as described (19). ChIP was performed using the Auto ChIP protein A kit (Diagenode) in the SX-8G IP-Star Compact Automated System (Diagenode) following manufacturer's instructions. Briefly, 30 μg of DNA and 2 μg of ER antibody, 3 μg of KDM3A antibody, 1 μg of H3K9me1/2 antibodies and equal amounts of isotype control antibodies were used for each ChIP reaction which comprised of 2-h antibody coating and 10-h immunoprecipitation incubation periods (antibody details: Supplementary Table S2). Following ChIP, eluted DNA and input samples taken from the original sonicated sample were subject to cross-link reversal and qPCR using primers specific to *pS2* and *GREB1* promoter elements and *CCND1, MYC* and *XBP1* distal enhancer elements (primer sequences: Supplementary Table S4). Data were calculated as % input (as described in (19)). Data were presented as the average fold difference of % input between different experimental arms (detailed in figure legends) of at least three independent experiments.

### RNAi rescue

RNAi rescue experiments were performed using pLenti-V5-KDM3A and the demethylase-inactive pLenti-V5-KDM3A_H1120G/D1122N_ plasmids. The pLenti constructs were generated by cloning the previously described pCMV-HA-KDM3A and pCMV-HA-KDM3A_H1120G/D1122N_ (26) plasmids into the pLenti6 backbone upstream and in-frame with the V5-tag via the pENTR shuttling vector using the pENTR/D-TOPO kit (Invitrogen) following manufacturer's instructions. Two silent (codon switch) mutations were introduced into the siKDM3A-B target sequence within both pLenti6-V5-KDM3A and pLenti6-V5-KDM3A_H1120G/D1122N_ plasmids by site directed mutagenesis using the Quickchange II kit (Agilent) incorporating the following primer sequences; F: 5′-GGGAATAAAGGCAAACTGCCCCTGCTCAAACAGGCAATTCAAAC-3′ and R: 5′-GTTTGAATTGCCTGTTTGAGCAGGGGCAGTTTGCCTTTATTCCC-3′ to allow ectopic expression of KDM3A in cells depleted of endogenous KDM3A.

To assess demethylase activities of KDM3A and KDM3A_H1120G/D1122N_, both plasmids were transfected into HEK293T cells and subject to immunofluorescence using H3K9me1/me2 methylation state-specific antibodies. Briefly, HEK293 cells plated onto sterile 22 mm × 22 mm coverslips and transfected for 48 h with 0.5 μg pLenti6-V5-KDM3A plasmids using TransIT-LT1 reagent (Mirus Bio) were fixed in 4% paraformaldehyde for 20 min at room temperature prior to consecutive incubations in 0.1% Triton (Sigma) for 10 min and 4% goat serum (DAKO) for 30 min at room temperature. Mixtures containing V5 and either H3K9me1/2/3-specific antibodies or isotype controls diluted in 4% goat serum to a final concentration of 1:1000 and 1:500, respectively (antibody details: Supplementary Table S3), were added to cells and incubated overnight at 4°C. A secondary antibody only control sample was also prepared by adding 4% goat serum to cells prior to overnight incubation. The next day, coverslips were washed in phosphate buffered saline (PBS) and secondary antibody mixes (prepared to a final dilution of 1:500 in 4% goat serum) were added for 1 at room temperature (antibody details: Supplementary Table S3) prior to washing in PBS and addition of Vectorshield DAPI mounting media (Vector Laboratories). Coverslips were mounted on microscope slides and analysed using a Leica DMR microscope system (Leica Microsystems). Once it was confirmed that neither the isotype control or secondary antibody stained cells showed any immunofluorescence, representative images of DAPI stained cells showing ectopic expression of KDM3A proteins (as indicated by V5 staining) and H3K9me1/me2 methylation were captured using SPOT Advanced software (Spot Imaging).

In preparation for RNAi rescue experiments, the pLenti6-V5-KDM3A plasmids were individually packaged into lentivirus in HEK293T cells using the ViraPower Lentiviral Packaging Mix (Invitrogen) following manufacturer's instructions. Virus was concentrated by ultracentrifugation at 26 500 x g for 2 h at 4°C and re-suspended in 1 ml of RPMI-1640 media ready for transduction into KDM3A-depleted MCF-7 cells. Briefly, MCF-7 cells were plated onto 12-well microtitre plates (Corning) and transfected with 25 nM siKDM3A-B for 2 h prior to the addition of KDM3A or KDM3A_H1120G/D1122N_ lentivirus. Parallel experiments were also performed in which MCF-7 cells were transfected with siSCR or siKDM3A-B but not transduced with lentivirus so that knockdown efficiency and relative ER-target gene expression could be assessed. Cells were grown for 65 h prior to RNA and protein extraction using TRIzol following manufacturer's instructions. cDNA was generated from extracted RNA and analysed by qPCR for KDM3A and ER-target gene expression (primer sequences: Supplementary Table S4). Gene expression data from each experiment was expressed relative to the expression measured in siSCR non-transduced MCF-7 cells and data were combined from at least three independent experiments. Protein extracted from RNAi rescue experiments was assessed by western blot analysis to confirm equal ectopic KDM3A expression.

### Cell growth analysis

Cell growth was assessed in siRNA-transfected MCF-7, T47D and MMU2 cells using cell counts, a BrdU ELISA assay (Roche) and the Incucyte Zoom live cell imager (Essen Bioscience). In each case MCF-7 and T47D cells were grown in phenol red-free RPMI-1640 media supplemented with 10% serum stripped FCS, 10 nM E_2_ and 1% penicillin/streptomycin.

For cell counts, siRNA-transfected MCF-7 and T47D cells were grown for 96 h on six-well plates prior to manual counting using a haemocytometer, MMU2 cells were grown for 72 h. Data were presented as the average fold difference in cell number relative to siSCR-transfected cells from three independent experiments. For phenotypic rescue analysis, RNAi rescue experiments were performed as previously described except cells were incubated for 156 h prior to cell counting. Data were presented as the mean cell number from three independent experiments.

The BrdU ELISA assay was performed following manufacturer's instructions (Roche). Briefly, cells were transfected with siRNA and grown for 72 h in 96-well plates prior to the addition of BrdU labelling solution. Cells were incubated for 16 h to allow BrdU incorporation and then the cells were washed, fixed and stained using a BrdU antibody conjugate. Cells were incubated for 90 min prior to staining using the BrdU substrate solution for 10 min before the reaction was stopped by the addition of 1 M H_2_SO_4_. The absorbance at 450 nm of each well was measured using the Bio-Rad 680 ELISA reader as an indication of BrdU incorporation. Data were calculated for each experimental arm as the mean absorbance from a minimum of five wells and was presented as the mean fold difference in absorbance relative to siSCR transfected cells from 3 independent experiments.

The Incucyte Zoom live cell imager was used to assess relative cell confluence between siRNA transfected MCF-7, T47D, MMU2 and MCF-10A cells. MMU2 cells and MCF-10A cells were grown in their respective normal growth media. Briefly, cells were transfected in 12-well plates and images from nine fields per well were taken every 6 h over 54 h (MCF-10A and MCF-7) 60 (MMU2) and 84 h (T47D) by the Incucyte Zoom live cell imager. The difference in incubation times between cell lines was dependent on differing cell growth rates of siSCR transfected cells. The Incucyte Zoom software package was trained to identify cells from each cell line and measure the % confluence of each well. Cell confluence was normalized for each well at the 0 time point and the relative change in cell confluence (hence growth) was calculated for each time point thereafter. Data were presented as the mean relative cell confluence at each time point from at least three independent experiments.

### Cell cycle analysis

Transfected MCF-7 and T47D cells were grown for 96 h in phenol red-free RPMI-1640 media supplemented with 10% serum-stripped FCS, 10 nM E_2_ and 1% penicillin/streptomycin prior to growth media being collected and cells being trypsinized. Growth media and trypsinized cells were combined, pelleted and washed in PBS before re-suspension in 100 μl citrate buffer (0.25 M Sucrose, 40 mM sodium citrate, pH 7.6). DNA staining and lysis buffer (20 μg/ml propidium iodide (Sigma), 0.5% NP-40 (Calbiochem), 250 μg/ml RNaseA (Qiagen) 0.5 mM ethylenediaminetetraacetic acid (EDTA), in PBS) was prepared and 400 μl added to the cell suspension prior to incubation overnight in the dark at 4°C. Following incubation, 10 000 propidium iodide-stained cells were measured using the FACScan (Becton Dickenson) together with CellQuest software (Beckton Dickenson) and analysed using Cyflogic software (CyFlo Ltd). The percentage of cells in each cell cycle phase was determined and data presented as the mean percentage of cells from three independent experiments.

## RESULTS

### KDM3A is required for ER signalling

**Figure 1.**
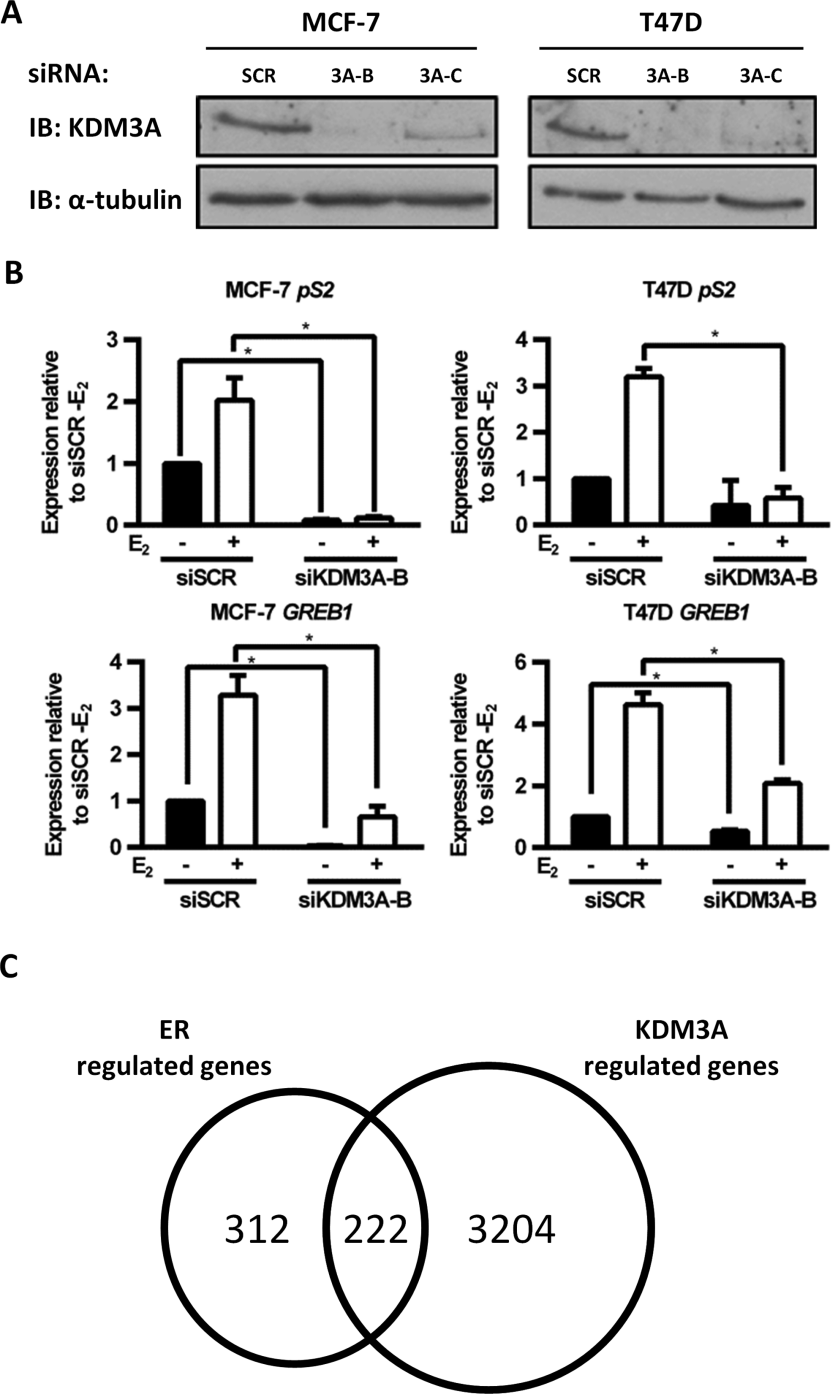
KDM3A is required for ER signalling. (**A**) MCF-7 and T47D cells were transiently transfected with a control siRNA (siSCR) or two independent KDM3A targeting siRNAs (siKDM3A-B and siKDM3A-C) and grown for 72 h prior to western analysis using antibodies specific to KDM3A and α-tubulin. α-Tubulin expression was used to confirm equal amounts of total protein between samples. (**B**) MCF-7 and T47D cell lines were subject to transient transfection with either siSCR or siKDM3A-B siRNAs and grown in steroid-depleted medium for 48 h prior to 4 h treatment with vehicle or 10 nM E_2_ and subsequent RNA extraction. Resultant cDNA was analysed for *pS2* and *GREB1* expression by qPCR. Data are the average of three independent experiments ± SEM. Gene expression is shown relative to that measured in vehicle treated siSCR transfected cells. *P-*values were determined by Student's *t*-test (* denotes *P* < 0.05). (**C**) MCF-7 cells were treated as in (B) prior to RNA extraction and hybridization to an Illumina HT12.2 Bead CHIP array. The number of ER-regulated genes (defined as genes significantly (*P* < 0.05) up- or down-regulated 1.5-fold by E_2_ stimulation in siSCR transfected cells (*n* = 534)), and the number of KDM3A-regulated genes (defined as genes significantly up- or down-regulated 1.5-fold by KDM3A knockdown compared to siSCR controls in E_2_ stimulated conditions (*n* = 3426)), was determined. E_2_-stimulated genes down-regulated by KDM3A knockdown or E_2_-repressed genes up-regulated by KDM3A knockdown were classified as E_2_-KDM3A-regulated genes (*n* = 222).

To identify HMT and HDM enzymes required for ER-mediated transcriptional regulation, an unbiased siRNA library screen was performed in which each of the human HMT and HDM enzymes were individually depleted in MCF-7 cells using a pool of three siRNAs. Receptor activity was assessed by measuring *pS2* gene expression (19). The screen identified that the HDM KDM3A was required for *pS2* expression (Supplementary Figure S1). Two individual siRNAs (siKDM3A-B and siKDM3A-C) were shown to robustly down-regulate protein expression of KDM3A compared to a scrambled control (siSCR) (Figure 1A) and both down-regulated expression of the ER-target genes *pS2* and *CCND1* in MCF-7, T47D, ZR751 and BT474 cells (Supplementary Figure S2A). KDM3A depletion using siKDM3A-B was also shown to significantly down-regulate the ER-target genes *pS2* and *GREB1* in the presence and absence of 10 nM β-estradiol (E_2_) (Figure 1B). Importantly, KDM3A knockdown did not affect ER protein expression, indicating that the observed effect on ER-target gene expression was not due to down-regulation of the receptor (Supplementary Figure S2B).

To interrogate the full extent of KDM3A regulation on ER signalling, we assessed basal and E_2_-stimulated global gene expression changes in KDM3A-depleted MCF-7 cells by microarray analysis. Briefly, cells were transfected in steroid-depleted conditions with either siSCR or siKDM3A-B for 48 h prior to treatment with vehicle or 10 nM E_2_ for 4 h before RNA extraction and hybridization to an Illumina HT12.2 Bead CHIP array. Successful KDM3A depletion was confirmed by qPCR (Supplementary Figure S2C).

Genes significantly up- or down-regulated 1.5-fold between vehicle and E_2_-treated siSCR transfected cells were deemed to be estrogen regulated (E_2_-regulated genes). We identified that 42% of E_2_-stimulated or repressed genes were respectively down- or up-regulated at least 1.5-fold in KDM3A-depleted cells compared to siSCR controls (termed E_2_-KDM3A regulated genes), indicating that KDM3A regulates a significant proportion of the ER transcriptome (Figure 1C). The expression of a large number of non ER-regulated genes was also affected by KDM3A knockdown, indicating that KDM3A also influences E_2_-independent transcription. A number of E_2_-stimulated genes down-regulated by KDM3A depletion are reported to play important roles in BCa progression. For example, *PIM1* is an ER regulated proto-oncogene that has been shown to play a role in BCa cell proliferation, migration and metastasis and is associated with high grade tumours (37,38); *KRT13* has been associated with BCa cell growth and metastasis (39) and *LOXL4* is an established promoter of cell invasion (40–42) (Supplementary Figure S2D). It should be noted that any E_2_-KDM3A regulated genes which are up- or down-regulated 1.5-fold post 4 h E_2_ treatment would not be identified in this analysis but may still play a role in BCa progression.

### KDM3A is required for ER recruitment to ER-responsive elements

**Figure 2.**
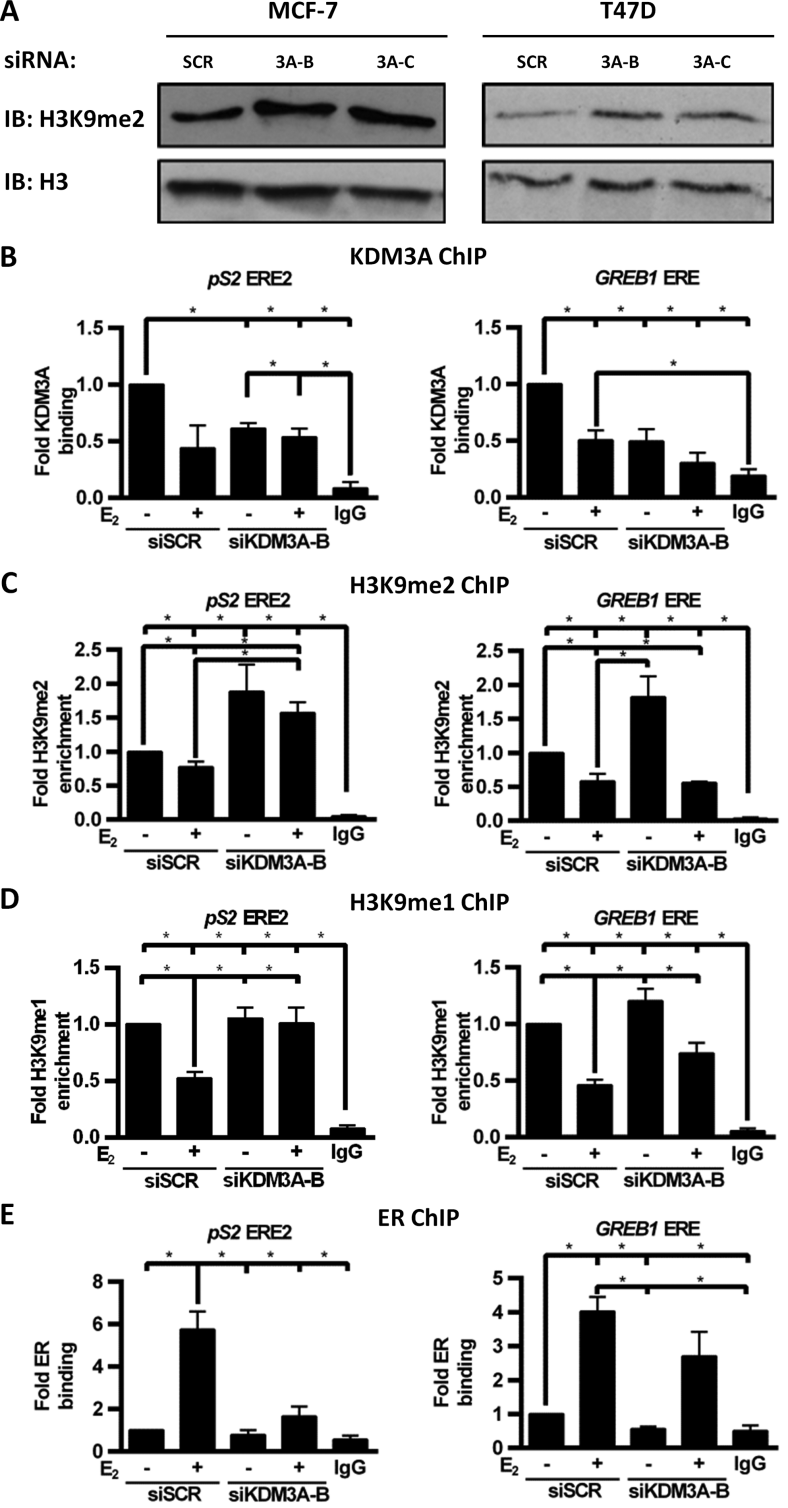
KDM3A regulates H3K9me1/2 demethylation and ER recruitment to target genes. (**A**) MCF-7 and T47D cells were transiently transfected with siSCR, siKDM3A-B or siKDM3A-C and grown for 48 h prior to western analysis using antibodies specific to H3K9me2 and histone H3. Histone H3 expression was used to confirm equal amounts of total histone protein between samples. (B–E) MCF-7 cells were transiently transfected with either siSCR or siKDM3A-B and grown for 24 h prior to treatment with vehicle or 10 nM E_2_ for 45 min followed by ChIP analysis using antibodies specific to KDM3A (**B**), H3K9me2 (**C**), H3K9me1 (**D**), ER (**E**) and isotype controls (IgG). Recruitment to ERE2 within the *pS2* promoter and an ERE within the *GREB1* promoter was assessed by qPCR. Data are an average of at least 3 independent experiments ± SEM and is expressed relative to the level of recruitment measured in vehicle treated siSCR transfected cells. *P*-values were determined by Turkey's multiple comparison test (* denotes *P* < 0.05).

We next sought to determine the mechanism by which KDM3A regulates ER signalling. KDM3A is a JmjC domain-containing HDM enzyme that removes transcriptionally repressive H3K9 mono- and di-methyl (H3K9me1/2) marks allowing DNA to be more accessible to transcription factors and the RNA polymerase machinery (10,11,23). We observed increased global H3K9me2 levels by western analysis in KDM3A-depleted MCF-7 and T47D cells confirming that the HDM activity of KDM3A is functional in ER-positive BCa cell lines (Figure 2A). We therefore hypothesized that KDM3A facilitates receptor activity by demethylating H3K9me1/2 at specific ER-target gene *cis*-regulatory elements.

To confirm E_2_-stimulated ER recruitment and H3K9me1/2 demethylation at ER-target gene *cis*-regulatory elements, we performed ChIP in MCF-7 cells treated with or without 10 nM E_2_ for 45 min. Using anti-ER, anti-H3K9me1 and anti-H3K9me2 antibodies and isotype controls, we confirmed that E_2_ stimulates ER recruitment and H3K9me1/2 demethylation at two estrogen response elements (EREs) within the promoter of *pS2* (ERE1 and ERE2) and one ERE within the promoter of *GREB1* (Supplementary Figure S3A–C). ChIP using an anti-KDM3A antibody indicated that KDM3A was present at EREs in E_2_-deprived conditions but was removed from chromatin following ligand stimulation (Supplementary Figure S3D). The presence of KDM3A at EREs under E_2_-deprived conditions correlates with the observation that KDM3A depletion reduces basal transcription of ER-regulated genes (Figure 1B; Supplementary Figure S2D).

To further define the role of KDM3A at ER-target gene promoters, H3K9 methylation and ER recruitment was assessed by ChIP in MCF-7 cells depleted of KDM3A. Knockdown of the enzyme by siKDM3A-B was confirmed at *pS2* and *GREB1* EREs under E_2_-deprived conditions (Figure 2B; Supplementary Figure S4A). Importantly, KDM3A depletion elevated the basal level of H3K9me2 at both *pS2* and *GREB1* EREs and abrogated H3K9me1/2 demethylation following hormone stimulation at *pS2* EREs, demonstrating that KDM3A is required to remove transcriptionally repressive marks at these loci (Figure 2C and D; Supplementary Figure S4B and C). In contrast, H3K9 demethylation remained evident upon E_2_ stimulation at the *GREB1* ERE despite KDM3A knockdown, suggesting that other HDMs which demethylate H3K9 may compensate for KDM3A depletion at this locus.

Importantly, reduced KDM3A levels attenuated ER recruitment to both *pS2* and *GREB1 cis-*regulatory elements in basal and estrogen-stimulated conditions (Figure 2E; Supplementary Figure S4D). The inhibition of ER recruitment upon E_2_ stimulation was more pronounced at *pS2* EREs than the *GREB1* ERE which correlates with the comparative effects of KDM3A depletion on H3K9me1/2 status at these sites. Inhibition of ER recruitment to *cis*-regulatory elements upon KDM3A depletion was also observed in T47D cells (Supplementary Figure S4E). Together, the data demonstrates that KDM3A regulates ER-mediated transcription by facilitating binding of the receptor to EREs via demethylation of H3K9me2/1.

Considering many ER–chromatin interactions occur in non-promoter regions we also investigated the effect of KDM3A knockdown on ER recruitment at distal enhancer elements. We observed that KDM3A knockdown abrogated ER recruitment to enhancer elements of the ER target genes *CCND1*, *MYC* and *XBP1*, demonstrating that the regulatory role of KDM3A on ER recruitment is not confined to target gene promoters and supporting the hypothesis that KDM3A is required for ER signalling (Supplementary Figure S5A–C).

### KDM3A catalytic activity is required for ER target gene expression

Compounds which target the catalytic activity of JmjC domain-containing KDM family members are being developed as potential cancer therapeutics (43,44). The validity of KDM3A as a potential therapeutic target in ER-positive BCa therefore relies on the catalytic activity of the enzyme driving ER signalling. The observation that KDM3A depletion concurrently abrogates H3K9me1/2 demethylation and ER recruitment suggests that KDM3A catalytic activity is required for successful ER signalling. To confirm this hypothesis, we assessed ER-target gene expression using RNAi-rescue experiments in MCF-7 cells depleted of endogenous KDM3A, but overexpressing siRNA-resistant wild-type or demethylase-inactive (KDM3A_H1120G/D1122N_ (26)) variants of the enzyme.

**Figure 3.**
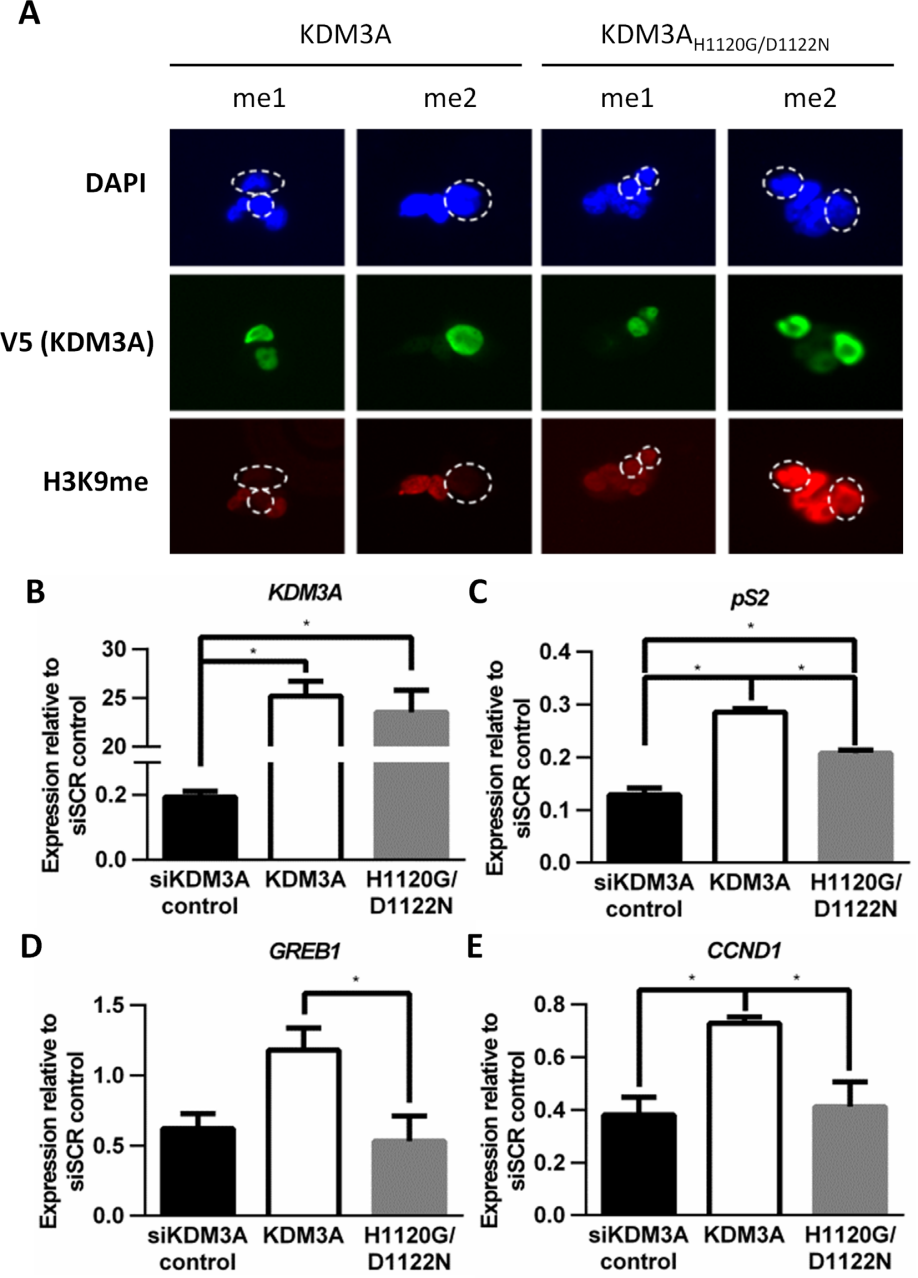
KDM3A catalytic activity is required for ER signalling. (**A**) HEK293 cells were transiently transfected with 0.5 μg of either V5-tagged wild-type KDM3A or demethylase-inactive KDM3A_H1120G/D1122N_–expressing plasmids and grown for 48 h prior to immunofluorescence using V5 (green), H3K9me1 and H3K9me2 (red) specific antibodies. Ectopic expression of wild type KDM3A (left panel) and KDM3A_H1120G/D1122N_ (right panel) was detected by V5 staining. Cells were counterstained using DAPI. The broken circles in DAPI and H3K9me1/2 fluorescent cell images indicate the position of cells ectopically expressing KDM3A. (B–E) MCF-7 cells were transiently transfected with siKDM3A-B and incubated for 2 h prior to transduction with lentivirus expressing either wild-type KDM3A or KDM3A_H1120G/D1122N_. Parallel control experiments were also performed in which MCF-7 cells were transfected with siSCR or siKDM3A-B. Cells were grown for 65 h prior to RNA extraction and resultant cDNA was analysed for *KDM3A* (**B**)*, pS2* (**C**)*, GREB1* (**D**) and *CCND1* (**E**) expression by qPCR. Data shown is for all siKDM3A transfected cells and is the average of at least three independent experiments ± SEM. Gene expression is shown relative to expression measured in siSCR control cells. *P*-values were determined by Turkey's multiple comparison test (* denotes *P* < 0.05).

Firstly, the respective catalytic activity of the KDM3A variants was confirmed by immuno-fluorescence using H3K9 methylation state-specific antibodies in HEK293 cells over-expressing either KDM3A or KDM3A_H1120G/D1122N_. Ectopically-expressed wild-type, but not the demethylase-inactive variant, was found to reduce global H3K9me1/2 (Figure 3A), while H3K9me3, which is not a demethylation target of KDM3A, remained unchanged (Supplementary Figure S6A). We next introduced KDM3A or KDM3A_H1120G/D1122N_ variants into siKDM3A-B-transfected MCF-7 cells via lentiviral transduction and measured *KDM3A, pS2, GREB1* and *CCND1* gene expression by qPCR. KDM3A mRNA levels were approximately 23-fold higher in KDM3A and KDM3A_H1120G/D1122N_ transduced cells than control siSCR transfected cells, demonstrating equal over-expression of KDM3A by both plasmids (Figure 3B). Equal KDM3A and KDM3A_H1120G/D1122N_ protein expression was also confirmed by western blot analysis (Supplementary Figure S6C). Importantly, expression of *pS2*, *GREB1* and *CCND1* was significantly elevated in KDM3A-transduced cells compared to cells transduced with KDM3A_H1120G/D1122N_ (Figure 3C–E). Expression of *GREB1* and *CCND1* demonstrated almost complete rescue in KDM3A-transduced cells to levels equivalent to the siSCR control, whereas KDM3A_H1120G/D1122N_ demonstrated no appreciable up-regulation of ER-target gene expression. This confirms that KDM3A catalytic activity is required for ER-regulated gene expression, and suggests that compounds targeting KDM3A catalytic activity may be effective in abrogating ER signalling.

### KDM3A is required for BCa cell growth

**Figure 4.**
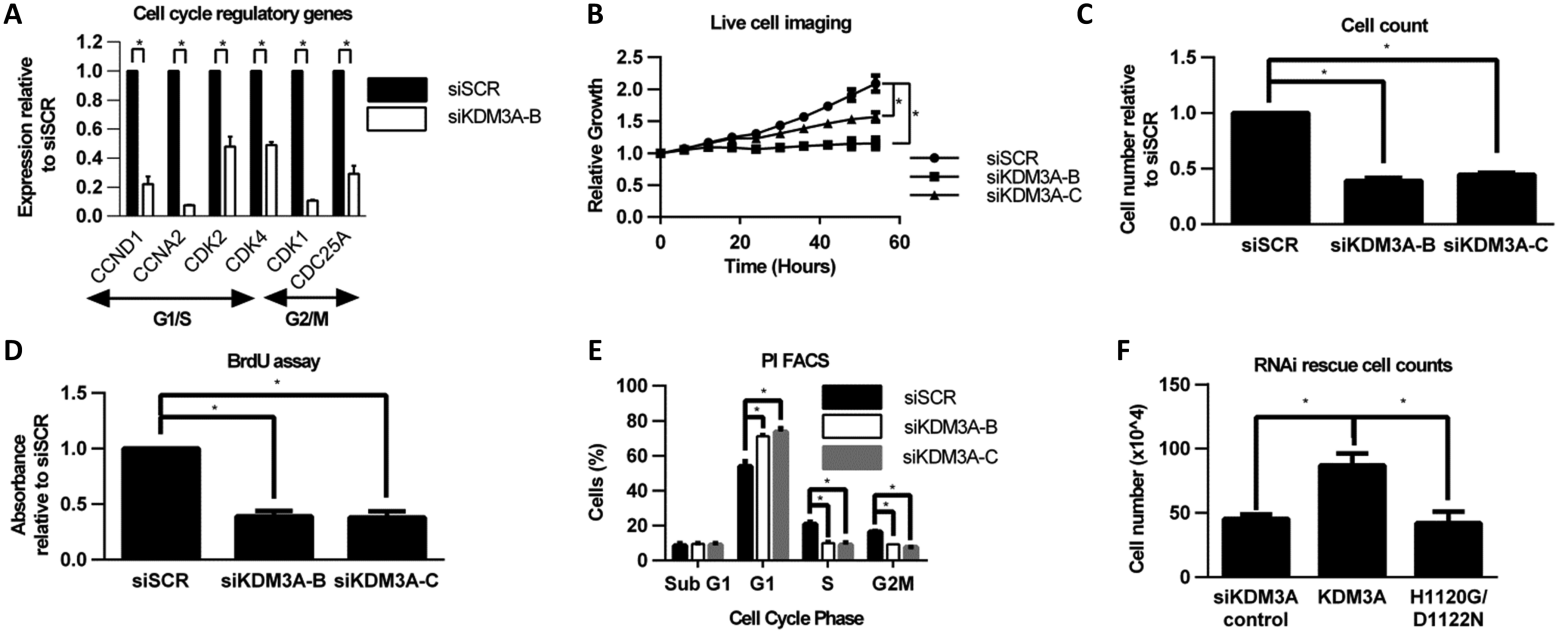
KDM3A is required for ER positive BCa cell growth. (**A**) Gene expression analysis of cell cycle regulatory genes (*CCND1, CCNA2, CDK2, CDK4, CDK1 and CDC25A*) between siSCR and siKDM3A-B-transfected E_2_-treated MCF-7 cells. Data are the average of three independent experiments ± SEM. Gene expression is shown relative to that measured in siSCR-transfected cells. (**B**) MCF-7 cells were transiently transfected with either siSCR, siKDM3A-B or siKDM3A-C and grown in steroid-depleted media containing 10 nM E_2_ for 54 h in the Incucyte Zoom live cell imager. Cell confluence was measured every 6 h. Data were normalized for each sample to the cell confluence measured at 0 h. Data are the average of three independent experiments ± SEM. (**C**) MCF-7 cells were treated as in (B) except grown for 96 h prior to cell counting. Data are the average of three independent experiments ± SEM and is expressed relative to the number of cells counted in siSCR transfected cells. (**D**) MCF-7 cells were treated as in (C) prior to analysis using a BrdU ELISA assay. Data are the average of three independent experiments ± SEM and is expressed relative to the absorbance for BrdU staining measured in siSCR transfected cells. (**E**) MCF-7 cells were treated as in (C) prior to harvesting for cell cycle analysis by propidium iodide flow cytometry. Data shows the % of cells in each phase of the cell cycle and is the average of three independent experiments ± SEM. (**F**) RNAi rescue experiment in MCF-7 cells (as described in Figure 3B–E) grown for 156 h prior to cell counting. Data shown is for all siKDM3A transfected cells and is the average of three independent experiments ± SEM. *P-*values were determined by Student's *t*-test (* denotes *P* < 0.05).

For KDM3A to be an effective therapeutic target, depletion of KDM3A must affect cancer cell growth and/or cell survival. To this end, we performed functional GO analysis on all KDM3A-regulated genes identified by our microarray to reveal which cellular processes are affected by KDM3A depletion. We identified a significant number of genes down-regulated by KDM3A knockdown that are required for cell cycle regulation and DNA replication (Supplementary Table S1). Validation of a subset of genes by qPCR in MCF-7 and T47D cells confirmed that KDM3A depletion down-regulates a number of cell cycle regulatory genes associated with both G1/S transition (*CCND1, CCNA2, CDK2, CDK4*) and G2/M transition (*CDK1*, *CDC25A*), suggesting that KDM3A is a positive regulator of BCa cell growth (Figure 4A; Supplementary Figure S7A).

To assess the phenotypic effect of KDM3A depletion on cell proliferation, we measured the growth of MCF-7 and T47D cells transfected with siSCR, siKDM3A-B or siKDM3A-C using live cell imaging, cell counts and a BrdU ELISA assay. In both BCa cell lines, proliferation was significantly reduced in cells depleted of KDM3A; confirming that KDM3A is required for normal cell growth (Figure 4B–D; Supplementary Figure S7B–D). Cell cycle analysis of siSCR, siKDM3A-B and siKDM3A-C-transfected MCF-7 and T47D cells using propidium iodide flow cytometry also demonstrated that KDM3A depletion caused an increase of cells in G1 phase of the cell cycle and respective decrease of cells in S and G2 phases, suggesting that KDM3A depletion causes G1 cell cycle arrest (Figure 4E; Supplementary Figure S7E). There was no increase in the proportion of cells in sub-G1 and no increase in caspase 3/7 activity in either cell line, demonstrating that KDM3A depletion is cytostatic rather than cytotoxic (Supplementary Figure S7F and G).

We next sought to confirm whether KDM3A catalytic activity is required for cell growth using RNAi-rescue experiments in MCF-7 cells. Consistent with the ER-target gene expression rescue experiments (Figure 3), proliferation was almost doubled in wild-type KDM3A-expressing cells compared to both KDM3A_H1120G/D1122N_-expressing and control KDM3A-depleted cells (Figure 4F), suggesting that KDM3A catalytic activity is required for MCF-7 cell growth.

A recent study demonstrated the use of a JmjC domain targeting compound (JIB-04) to inhibit prostate cancer and lung cancer growth but showed no detrimental effect on normal cell types (43). We also observed no impact on cell growth in the non-transformed breast epithelial cell line MCF-10A following KDM3A depletion, suggesting that compounds which affect KDM3A activity may be useful as tumour specific BCa therapies (Supplementary Figure S8).

### KDM3A is required for endocrine therapy-resistant cell growth

**Figure 5.**
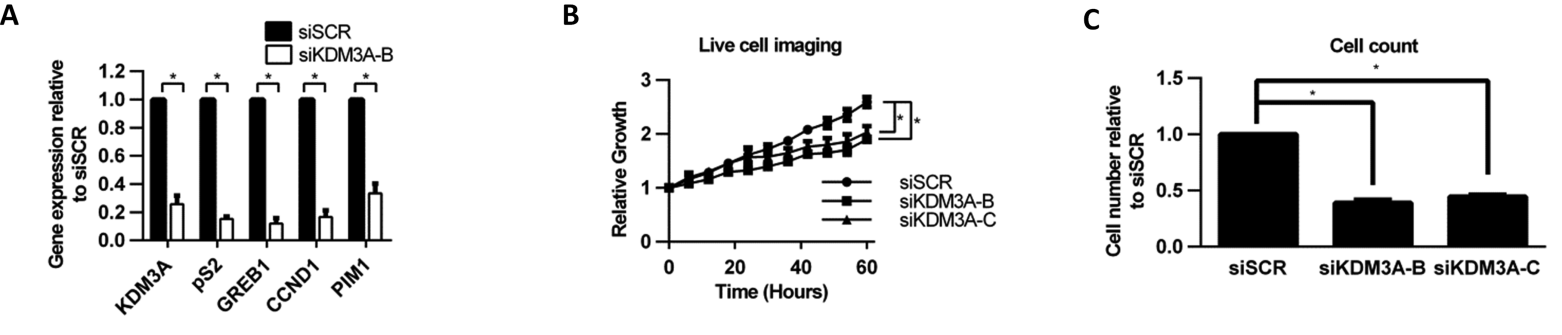
KDM3A is required for ER-target gene expression and cell growth in Tamoxifen-resistant cells. (**A**) MMU2 cells were transiently transfected with siSCR and siKDM3A-B and grown for 72 h prior to RNA extraction. Resultant cDNA was analysed for *KDM3A*, *pS2*, *GREB1*, *CCND1* and *PIM1* expression by qPCR. Data are the average of three independent experiments ± SEM. Gene expression is shown relative to that measured in siSCR transfected cells. (**B**) MMU2 cells were transiently transfected with siSCR, siKDM3A-B and siKDM3A-C and grown for 60 h in the Incucyte Zoom live cell imager. Cell confluence was measured every 6 hours. Data were normalized to cell confluence measured at 0 h. Data are the average of five independent experiments ± SEM. *P-*values were determined by Student's *t*-test (* denotes *P* < 0.05). (**C**) MMU2 cells were treated as in (B) except grown for 72 h prior to cell counting. Data are the average of three independent experiments ± SEM and is expressed relative to the number of cells counted in siSCR transfected cells.

Identifying novel therapeutic targets within the ER signalling cascade is essential to combat endocrine therapy-resistant disease. We therefore investigated the effect of KDM3A depletion on endocrine therapy-resistant BCa growth. The MCF-7 derived MMU2 cell line is a model of endocrine therapy-resistant disease that is ER-positive but can grow in E_2_-deprived conditions and is resistant to tamoxifen treatment (45). Although tamoxifen resistant, MMU2 cells still require ER expression for growth and ER target gene expression (Supplementary Figure S9A–C). We transfected MMU2 cells with either siSCR or siKDM3A-B and demonstrated that KDM3A depletion significantly down-regulates expression of the ER-target genes *pS2, GREB1, CCND1* and *PIM1* (Figure 5*A*). Western analysis confirmed successful KDM3A depletion in MMU2 cells (Supplementary Figure S9D). The data therefore indicates that KDM3A continues to regulate ER signalling in cells which have developed endocrine therapy resistance. Crucially, KDM3A depletion also reduced MMU2 cell proliferation, suggesting that therapeutics targeting KDM3A could potentially be used to treat endocrine therapy-resistant disease (Figure 5B and C).

## DISCUSSION

The suggested role of ER co-regulator proteins in both BCa development and acquisition of endocrine therapy-resistance have identified these enzymes as potentially important BCa therapeutic targets (3). Understanding the mechanism of regulation as well as the transcriptional and phenotypic effects of targeting these enzymes is therefore crucial in developing therapies which target their action.

Using an siRNA screen we identified that the HDM enzyme KDM3A was required for expression of the ER target gene *pS2*, and using global gene expression analysis showed that 42% of ER-regulated genes were also regulated by KDM3A. KDM3A has previously been implicated in regulating ER target-gene expression (18,46), however, this is the first study to our knowledge which has shown a significant transcriptome-wide role for KDM3A regulation in ER signalling. KDM3A is up-regulated during hypoxia by HIF-1α-mediated transcriptional activation and has been shown to be vital for hypoxia-mediated gene expression changes (26,27,47). There have been a number of studies which suggest significant interplay between hypoxia and ER signalling pathways and hypoxia has even been implicated in promoting hormone-independent breast tumour growth (48–51). Experiments in this study were not performed under hypoxic conditions and therefore the impact of KDM3A knockdown was not mediated by hypoxia. However, the significant impact on ER signalling by KDM3A knockdown does suggest that KDM3A activity may be a link between these two pathways, and could therefore be a promising target for both ER-positive and hormone-independent disease.

As well as showing the global impact of KDM3A knockdown on ER signalling we also identified E_2_-stimulated genes down-regulated by KDM3A knockdown that have important roles in BCa growth and metastasis (*PIM1, LOXL4* and *KRT13*), suggesting that KDM3A could play an important role in ER-positive BCa development (37–42). The identification of such genes could act as biomarkers of KDM3A dysregulation and could be used to assess the success of KDM3A targeting therapies in treating ER-positive BCa. KDM3A has also been implicated in neuroblastoma metastasis (52) and the HDM enzyme KDM4C has been shown to be a regulator of BCa lung metastasis and an activator of *LOXL2* expression (21). The regulatory role of these two HDM enzymes on the expression of pro-metastatic genes therefore suggests that dysregulated histone methylation may play an important role in BCa metastasis.

KDM3A removes transcriptionally repressive mono- and di-methyl marks from H3K9 and has been shown to be required for successful AR recruitment to AR-target gene promoters in prostate cancer cell lines upon ligand stimulation (23). Using ChIP, we showed that knockdown of KDM3A repressed H3K9 demethylation, and abrogated ER recruitment to *cis*-regulatory elements within the promoter regions of the ER-target genes *pS2* and *GREB1* and at distal enhancer elements of the ER-target genes *CCND1, MYC* and *XBP1*, indicating that KDM3A is also required for receptor recruitment in ER-positive BCa. Interestingly, the repressive effect of KDM3A knockdown on H3K9 demethylation and ER recruitment upon E_2_ stimulation was less pronounced at the *GREB1* promoter compared to *pS2*, indicating that multiple HDM enzymes may regulate some sites and could therefore compensate for KDM3A down-regulation at specific *cis*-regulatory elements. For example, the HDMs KDM1A and KDM4B which regulate ER transcriptional activity can both demethylate H3K9me2 and may therefore contribute to redundancy in epigenetic regulation of specific sites (18,19). Redundancy in epigenetic regulation of signalling pathways may suggest that pan-inhibitors of HDM enzymes could be ultimately more effective as cancer therapeutics than HDM family-, or enzyme-specific compounds.

We also observed that KDM3A was apparently removed from EREs after hormone stimulation. This was surprising considering that other ER co-regulators, such as KDM4B, are recruited to EREs following E_2_ stimulation (18,19,22,53,54). The cause of E_2_-stimulated removal of KDM3A is currently unclear but may be due to either epitope masking of the anti-KDM3A antibody by components of the active ER complex, or active removal once the *cis*-regulatory element is primed to enable recruitment of additional co-stimulatory proteins.

In order to determine the efficacy of KDM3A as a potential BCa therapeutic target we sought to identify phenotypic changes associated with KDM3A knockdown. GO analysis of KDM3A regulated genes in ER-positive BCa cells identified gene clusters associated with cell cycle regulation and DNA replication. A number of cell cycle regulatory genes associated with cell cycle progression were down-regulated by KDM3A knockdown. Consistent with this, knockdown of KDM3A reduced cell proliferation in both MCF-7 and T47D cell lines via a cytostatic mechanism. KDM3A depletion has previously been shown to reduce cell growth in a number of cancer contexts (25–28). Our data support the observation that KDM3A is important for cancer cell proliferation and the dramatic cytostatic effect of KDM3A depletion on BCa cell growth demonstrates that KDM3A would be an attractive BCa therapeutic target. KDM3A knockout mouse models are viable with only male germ cell defects and signs of early onset obesity (55–57). This fact, coupled with the observation that KDM3A knockdown had no effect on proliferation of the immortalized, but non-transformed, breast epithelial cell line MCF-10A, suggests that therapies targeting KDM3A may have minimal detrimental side-effects, which is an important consideration when validating potential therapeutic targets.

Pan- and selective-inhibitors of HDM enzymes are currently being developed which work by blocking the catalytic activity of their HDM targets (43,44). For example, the pan-inhibitor JIB-04 is an inhibitor of JmjC domain containing HDM enzymes which blocks Fe(II) binding. JIB-04 was shown to affect lung and prostate cancer cell growth without effecting normal, untransformed cell growth indicating that HDM targeting compounds may be cancer specific (43). As previously discussed, we have shown a potential cancer specific growth-inhibitory phenotype upon KDM3A depletion, indicating targeting KDM3A may be a useful cancer specific treatment. We have also shown that KDM3A catalytic activity is required for both ER signalling and ER-positive BCa cell growth indicating that compounds which target KDM3A catalytic activity may be useful as potential BCa therapies. Although no KDM3 family member-specific compounds are currently available, selective agents targeted to HDMs are being developed, supporting the concept that these proteins represent important therapeutic targets in cancer (44).

Finally, we assessed the effect of KDM3A knockdown on a model of endocrine therapy-resistant disease. It has been demonstrated that some endocrine therapy-resistant BCa models are disengaged from ER signalling (58), however, some are still reliant on ER expression for growth. The MMU2 cell line is an established model of therapy-resistant ER-positive BCa that was developed by continued long term culture of MCF-7 cells in 4-hydroxytamoxifen, an active tamoxifen metabolite (45). The ER signalling pathway is functional in these cells in the absence of ligand and in the presence of Tamoxifen (45) and ER expression is still required for cell growth. We have shown that KDM3A knockdown down-regulates ER target gene expression in MMU2 cells. This is significant as it demonstrates that KDM3A is an E_2_-independent regulator of ER signalling in endocrine therapy-resistant disease, and therefore may be a useful treatment to abrogate ER signalling in these cancer types. Crucially, KDM3A knockdown also inhibited MMU2 cell growth demonstrating an important phenotypic impact on endocrine therapy-resistant disease. The effect of KDM3A knockdown on MMU2 cell growth was not quite as robust as in MCF-7 and T47D cells suggesting that other HDM enzymes, for example KDM4B which has been shown to be a positive regulator of BCa cell growth and is overexpressed in BCa, may also play important roles in maintaining therapy-resistant cell growth. The effect of KDM3A knockdown in therapy-resistant disease therefore suggests that investigation of other HDM enzymes may provide further future therapeutic targets. Endocrine therapy has been very successful in treating ER-positive BCa, but resistance to these regimens remains problematic. Identifying targets, such as KDM3A, that have an effect on the maintenance of therapy-resistant BCa is therefore crucial in future management of the disease.

In summary, we have established that KDM3A is essential for ER signalling and that KDM3A regulates receptor-target gene transcription by controlling demethylation of H3K9me1/me2 at *cis*-regulatory elements. We have established that KDM3A regulates the expression of a number of genes involved in BCa progression and proliferation and have convincingly demonstrated that KDM3A is required for ER positive BCa cell growth. KDM3A was also required for cell growth in a model for endocrine therapy-resistant disease. Importantly, we have confirmed that the catalytic activity of KDM3A is required for both ER signalling and BCa cell growth and therefore suggest that developing HDM catalytic-targeting agents would be useful BCa therapeutics. KDM3A is expressed in BCa tissue and it has been reported following study of The Cancer Genome Atlas (TCGA) human breast cancer database (*n* = 729) that patients with BCa expressing high levels of KDM3A had a 3-fold increased risk of death than those with normal or low KDM3A expressing disease (43). The effect of KDM3A depletion detailed in this study therefore indicates that KDM3A is a promising BCa therapeutic target.

## SUPPLEMENTARY DATA

Supplementary Data are available at NAR Online.

SUPPLEMENTARY DATA
