# Supplementary material for: The histone demethylase enzyme KDM3A is a key estrogen receptor regulator in breast cancer
Source: Nucleic Acids Res. 2014 Dec 8;43(1):196–207. doi: 10.1093/nar/gku1298 (PMC4288188; doi:10.1093/nar/gku1298)
Supplement: SUPPLEMENTARY DATA [file supp_43_1_196__index.html]

The histone demethylase enzyme KDM3A is a key estrogen receptor regulator in breast cancer — SUPPLEMENTARY DATA 

# The histone demethylase enzyme KDM3A is a key estrogen receptor regulator in breast cancer

## SUPPLEMENTARY DATA

**Files in this Data Supplement:**

- SUPPLEMENTARY DATA
