# Supplementary material for: The histone demethylase enzyme KDM3A is a key estrogen receptor regulator in breast cancer
Source: Nucleic Acids Res. 2014 Dec 8;43(1):196–207. doi: 10.1093/nar/gku1298 (PMC4288188; doi:10.1093/nar/gku1298)
Supplement: SUPPLEMENTARY DATA [file supp_gku1298_nar-03081-x-2014-File007.pdf]

Supplementary Information

To accompany Wade *et al.*, 'The histone demethylase enzyme KDM3A is a key estrogen receptor regulator in breast cancer'

Supplementary Figure Legends

Supplementary Figure S1. MCF-7 cells were transfected with either siSCR or a pool of three individual siRNAs per HMT and HDM enzyme studied and grown in steroid depleted medium in the presence or absence of 10 nM E_2 for 72 hours prior to RNA extraction. Resultant cDNA was assessed for *pS2* expression by qPCR. Control experiments were also performed in which cells were transfected with either an ER (siER) or pS2 (sipS2) siRNA to confirm successful *pS2* down-regulation. Data is the average of 3 independent experiments +/- SEM. *pS2* expression is shown relative to that measured in vehicle treated siSCR transfected cells. KDM3A is highlighted with a red *.

Supplementary Figure S2. (*A*) MCF-7, T47D, ZR751 and BT474 cells were transiently transfected with siSCR, siKDM3A-B or siKDM3A-C siRNAs and grown for 72 hours prior to RNA extraction. Resultant cDNA was assessed for *pS2* and *CCND1* expression by qPCR. Data is the average of 3 independent experiments +/- SEM. *P* values were determined by Students *t* test (* denotes $p<0.05$ between siKDM3A-transfected cells and siSCR-transfected cells) (*B*) MCF-7 cells transfected with siSCR, siKDM3A-A (which had off target effects and was therefore not used in any further experiments), siKDM3A-B or siKDM3A-C siRNAs were grown for 72 hours prior to western analysis using ER and α-tubulin-specific antibodies. (*C*) MCF-7 cells were subject to transient transfection with either siSCR or siKDM3A-B and grown in steroid-depleted medium for 48 hours prior to 4 hour treatment with vehicle or 10 nM E_2 and RNA extraction. Resultant cDNA was analysed for *KDM3A* expression by qPCR. Data is the average of 3 independent experiments +/- SEM. Gene expression is shown relative to expression measured in vehicle-treated siSCR transfected cells. *P* values were determined by Students *t* test (* denotes $p<0.05$). (*D*) E_2 up-regulated genes identified by gene expression microarray that are down-regulated by KDM3A depletion and are linked to BCa progression. Data is the average of 2 independent experiments +/- SD. Gene expression is shown relative to that measured in vehicle treated siSCR transfected cells. *P* values were determined by Students *t* test (* denotes $p<0.05$).

Supplementary Figure S3. MCF-7 cells were grown for 48 hours prior to treatment with vehicle or 10 nM E_2 for 45 minutes followed by ChIP analysis using antibodies specific to (*A*) ER, (*B*) H3K9me1, (*C*) H3K9me2, (*D*) KDM3A, and isotype controls (IgG). Recruitment to EREs within the *pS2* and *GREB1* promoters was assessed by qPCR. Data is an average of 3 independent experiments +/- SEM and is expressed relative to the level of recruitment measured in vehicle treated siSCR transfected cells. *P* values were determined by Turkey's multiple comparison test (* denotes $p<0.05$).

Supplementary Figure S4. (*A*-*D*) MCF-7 cells were transiently transfected with siSCR and siKDM3A-B and grown for 24 hours prior to treatment with vehicle or 10 nM E_2 for 45 minutes followed by ChIP analysis using antibodies specific to (*A*) KDM3A, (*B*) H3K9me1, (*C*) H3K9me2, (*D*) ER, and isotype controls (IgG). Recruitment to *pS2* ERE1 and a region within the *pS2* promoter which the ER does not bind (*pS2* control region) was assessed by qPCR. Data is an average of at least 3 independent experiments +/- SEM and is expressed relative to the level of recruitment measured in vehicle

treated siSCR transfected cells. (*E*) T47D cells were treated as above and assessed by ChIP using an ER-specific antibody and an isotype control (IgG). Recruitment to EREs within the *pS2* and *GREB1* promoters was assessed by qPCR. Data is an average of 3 independent experiments +/- SEM and is expressed relative to the level of recruitment measured in vehicle treated siSCR transfected cells. *P* values were determined by Turkey's multiple comparison test (* denotes p<0.05).

Supplementary Figure S5. (*A-C*) MCF-7 cells were transiently transfected with siSCR and siKDM3A-B and grown for 24 hours prior to treatment with vehicle or 10 nM E_2 for 45 minutes followed by ChIP analysis using antibodies specific to the ER and an isotype control (IgG). Recruitment to distal ER enhancer elements positioned outside the promoter regions of the ER target genes *CCND1, MYC,* and *XBP1* was assessed by qPCR. Data is the average of 2 independent experiments +/- SEM and is expressed relative to the level of recruitment measured in vehicle treated siSCR transfected cells. *P* values were determined by Turkey's multiple comparison test (* denotes p<0.05).

Supplementary Figure S6. (*A*) HEK293T cells were transiently transfected with 0.5 µg of V5 tagged wild type KDM3A (left panel) or V5 tagged $KDM3A_{H1120G/D1122N}$ (right panel) and grown for 48 hours prior to analysis by immuno-fluorescence using V5 (green) and H3K9me3 (red) specific antibodies. Cells were counterstained using DAPI. The broken circles in DAPI and H3K9me3 fluorescent cell images indicate the position of cells ectopically-expressing KDM3A variants. (*B*) The position of 2 codon-switch mutations (red) within the siKDM3A-B target sequence introduced into KDM3A plasmids by site-directed mutagenesis. (*C*) MCF-7 cells were transiently transfected with siKDM3A-B prior to transduction with lentivirus containing either KDM3A or $KDM3A_{H1120G/D1122N}$ 2 hours later. Cells were grown for 65 hours prior to western analysis using KDM3A and α-tubulin specific antibodies. α-tubulin expression was used to confirm equal amounts of total protein between samples.

Supplementary Figure S7. (*A*) Validation of cell cycle regulatory gene expression in KDM3A-depleted T47D cells. Cells were subject to transient transfection with either siSCR or siKDM3A-B siRNAs and grown in steroid-depleted medium for 48 hours prior to 4 hour treatment with 10 nM E_2 and subsequent RNA extraction. Resultant cDNA was analysed for *CDK1, CDK4, CCNA2*, *CDC25A* and *CCND1* expression by qPCR. Data is the average of 3 independent experiments +/- SEM. Gene expression is shown relative to that measured in siSCR-transfected cells. (*B*) T47D cells were transiently transfected with siSCR, siKDM3A-B or siKDM3A-C and grown in steroid-depleted media containing 10 nM E_2 for 84 hours in the Incucyte Zoom live cell imager. Cell confluence was measured every 6 hours. Data was normalised for each sample to the cell confluence measured at 0 hours. Data is the average of 3 independent experiments +/- SEM. (*C*) T47D cells were treated as in (B) except grown for 96 hours prior to cell counting. Data is the average of 3 independent experiments +/- SEM and is expressed relative to the number of cells counted in siSCR transfected cells. (*D*) T47D cells were treated as in (C) prior to analysis using a BrdU ELISA assay. Data is the average of 3 independent experiments +/- SEM and is expressed relative to the absorbance for BrdU staining measured in siSCR-transfected cells. (*E*) T47D cells were treated as in (C) prior to harvesting for cell cycle analysis by propidium-iodide flow cytometry. Data shows the % of cells in each phase of the cell cycle and is the average of 3 independent experiments +/- SEM. (*F* and *G*) MCF-7 and T47D cells were transiently transfected with either siSCR, siKDM3A-B or siKDM3A-C siRNAs and grown for 96 hours prior to harvesting for a caspase 3/7 ELISA assay to detect the relative amount of cells undergoing apoptosis. Data is the average of 3 independent experiments +/- SEM and is expressed

relative to the amount of caspase 3/7 activity in siSCR-transfected cells. *P* values were determined by Students *t* test (* denotes p<0.05).

Supplementary Figure S8. (*A*) MCF-10A cells were transiently transfected with siSCR, siKDM3A-B or siKDM3A-C and grown for 54 hours in the Incucyte Zoom live cell imager. Cell confluence was measured every 4 hours. Data was normalised for each sample to the cell confluence measured at 0 hours. Data is the average of 3 independent experiments +/- standard error. (B) Western analysis of transfected cells shows successful knockdown of KDM3A over this time period by siKDM3A-B and siKDM3A-C.

Supplementary Figure S9. (*A–C*) MMU2 cells were transiently transfected with siSCR and siER siRNAs and grown for 72 hours prior to western analysis using ER and α-tubulin specific antibodies (*A*), RNA extraction, cDNA generation and subsequent analysis of the ER target genes *pS2, GREB1,* and *CCND1* by qPCR (*B*) and cell counts (*C*). Gene expression data is the average of 3 independent experiments +/- SEM and is shown relative to expression measured in siSCR-transfected cells. Cell count data is the average of 3 independent experiments +/- SEM and is shown relative to cell numbers counted in siSCR-transfected cells. *P* values were determined by Students *t* test (* denotes p<0.05). (*D*) MMU2 cells were transiently transfected with siSCR, siKDM3A-B and siKDM3A-C siRNAs and grown for 60 hours before western analysis using KDM3A and α-tubulin specific antibodies.

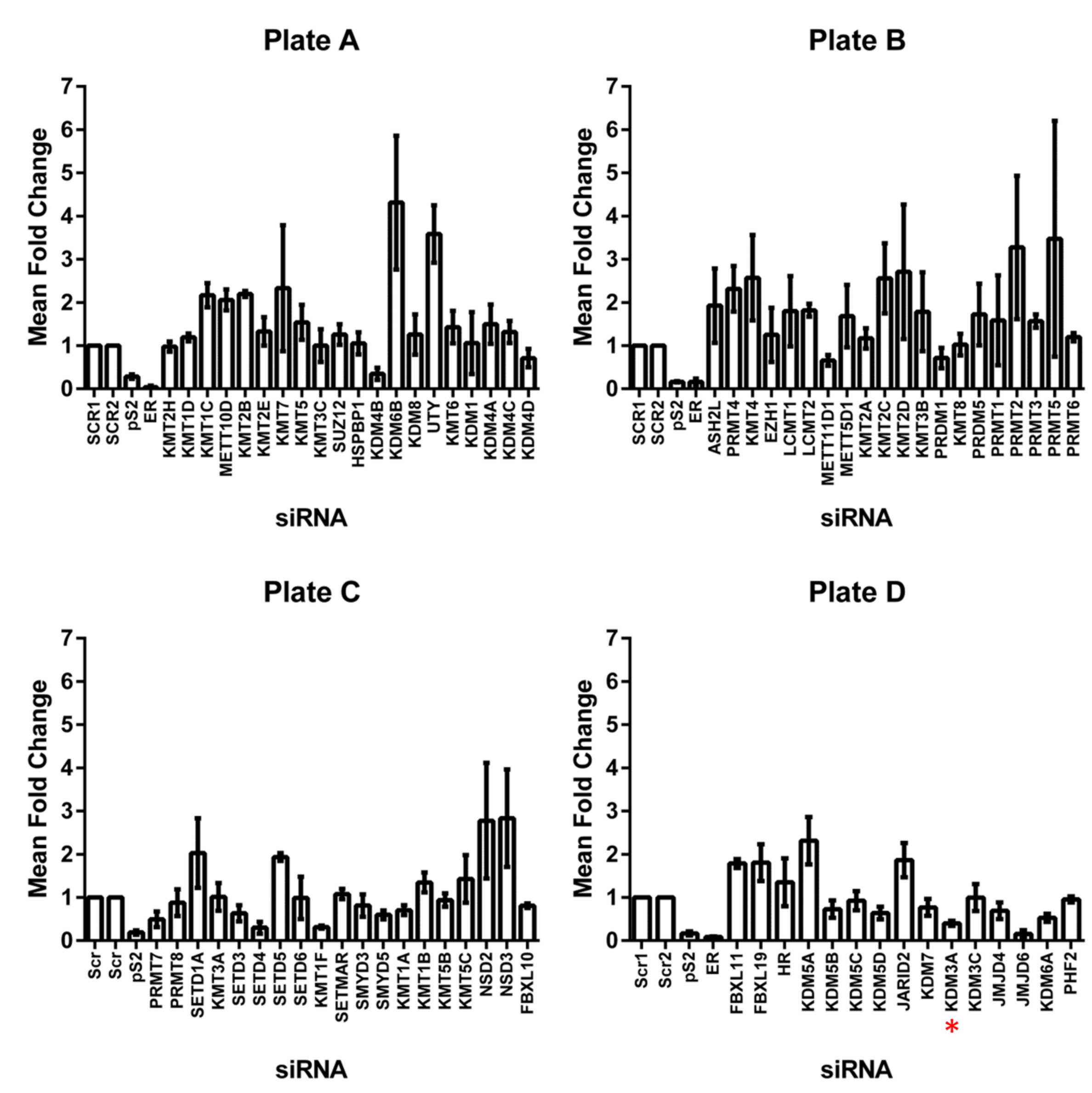

Supplementary Figure S1

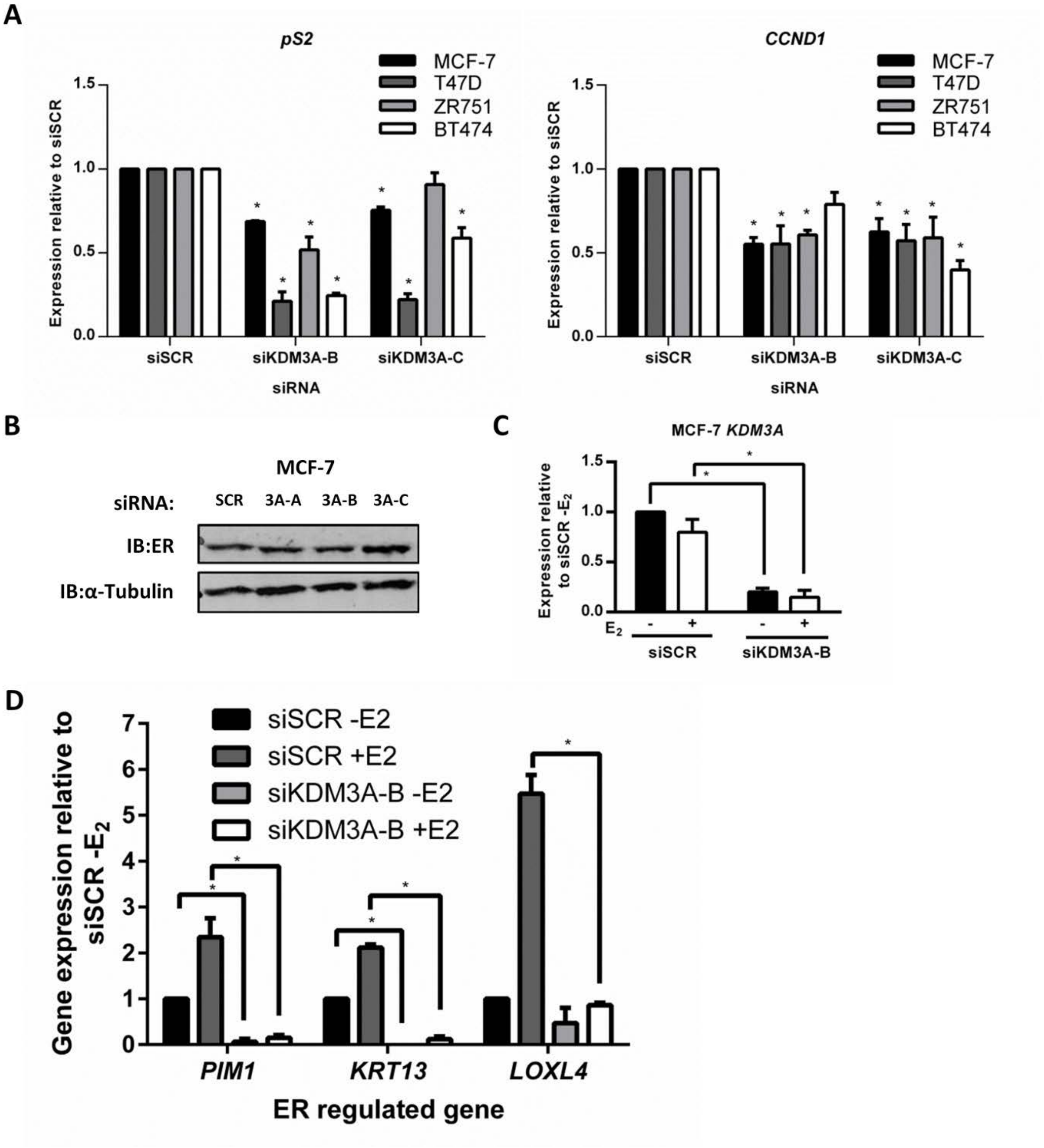

Supplementary Figure S2

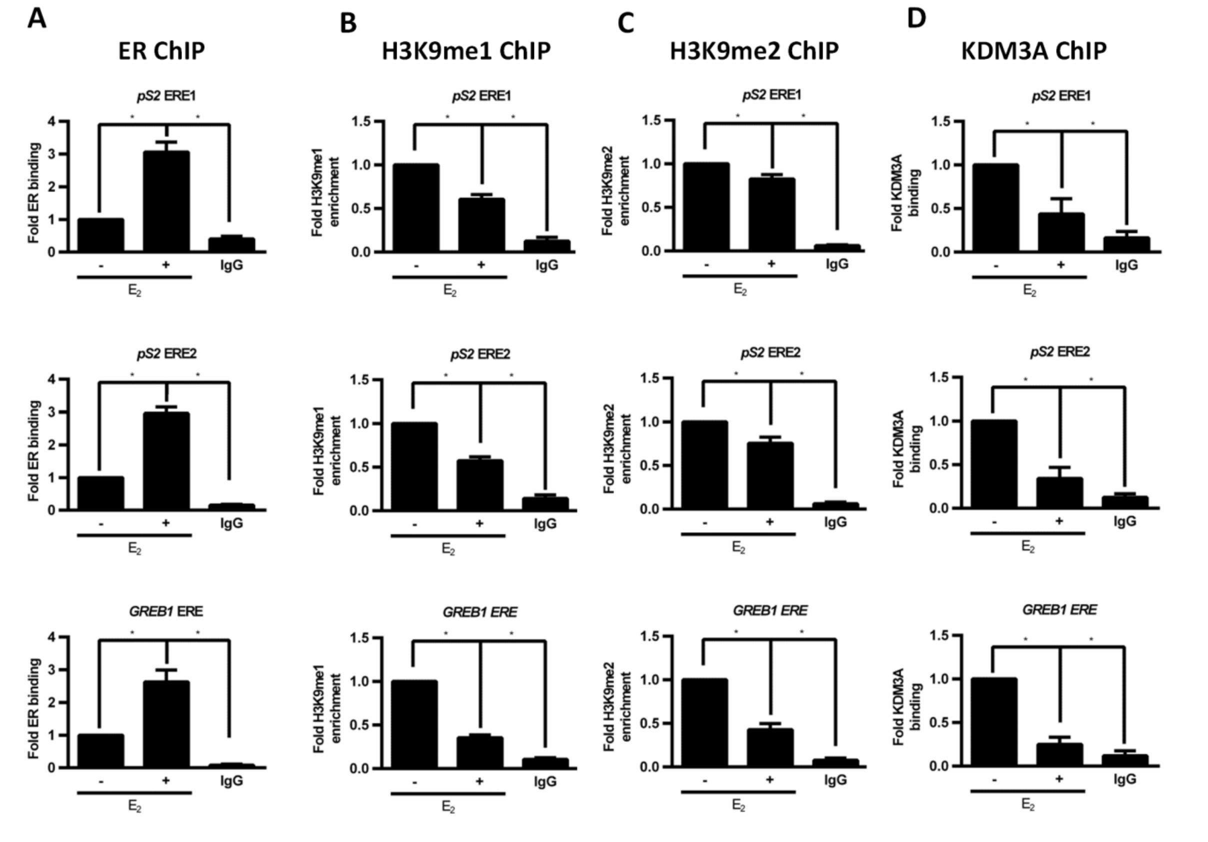

Supplementary Figure S3

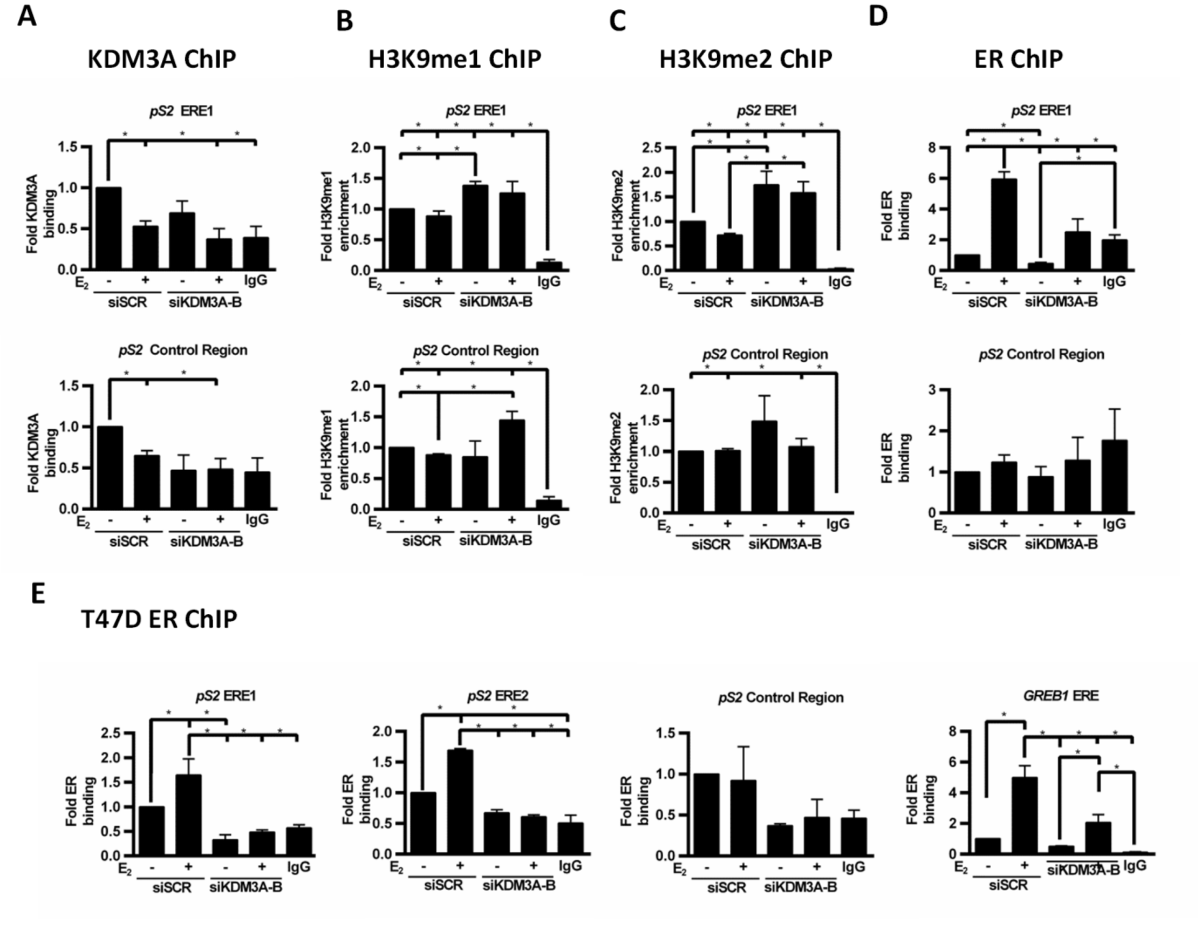

Supplementary Figure S4

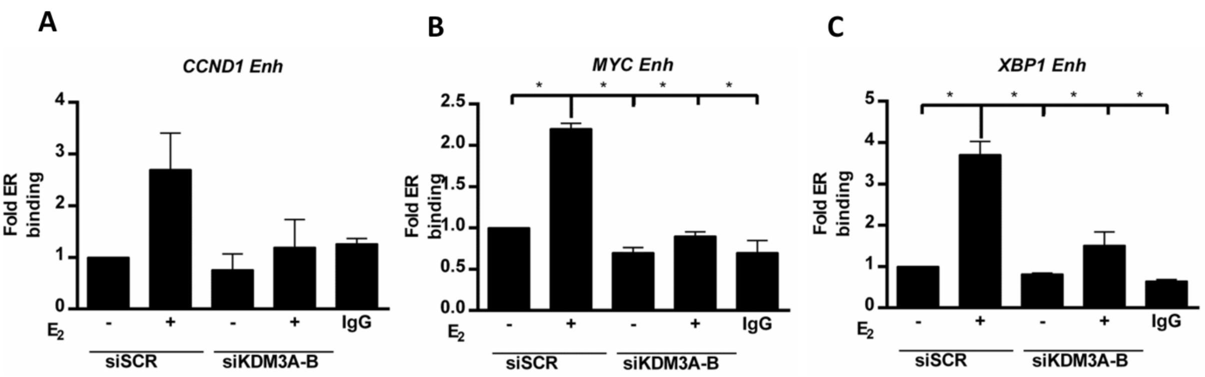

Supplementary Figure S5

A

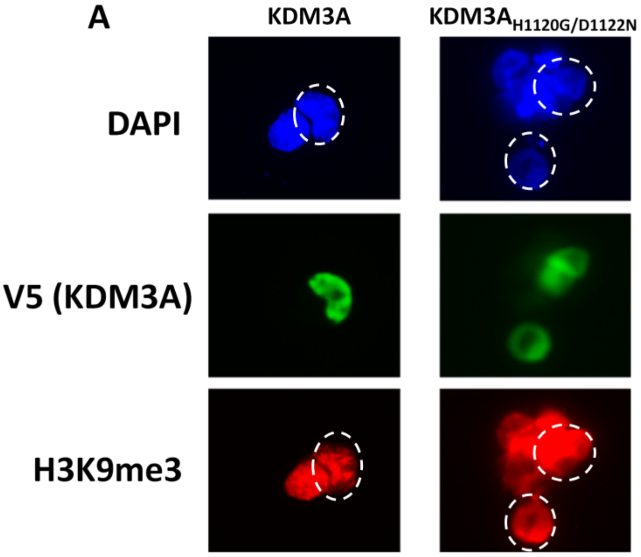

B

siKDM3A-B target sequence - CAAACTGCCCTTGTTCAAA
KDM3A variant siKDM3A-B target sequence - CAAACTGCCCCTGCTCAAA

C

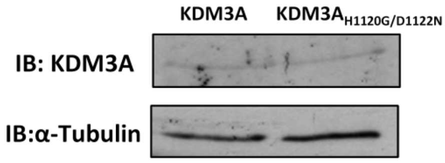

Supplementary Figure S6

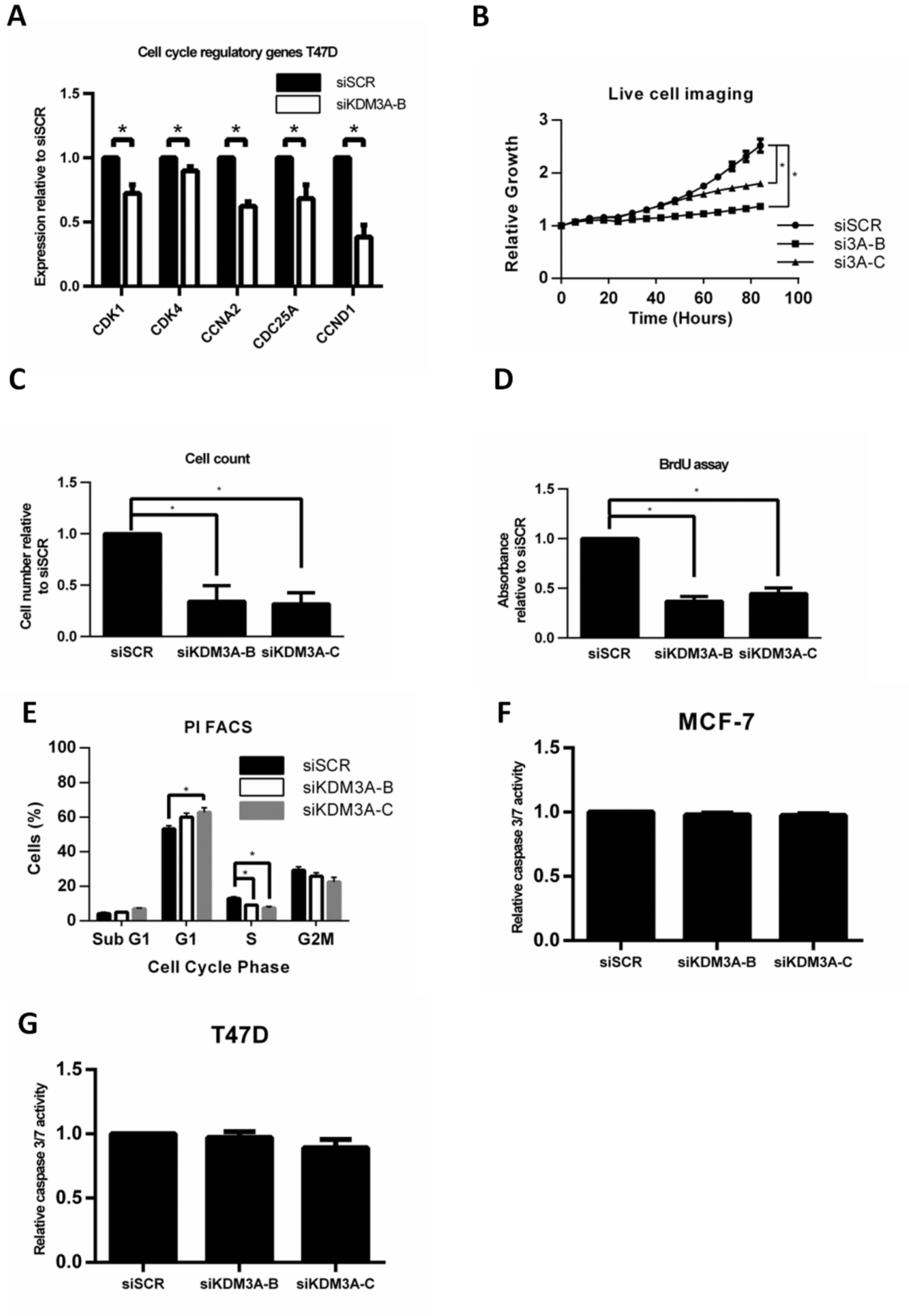

Supplementary Figure S7

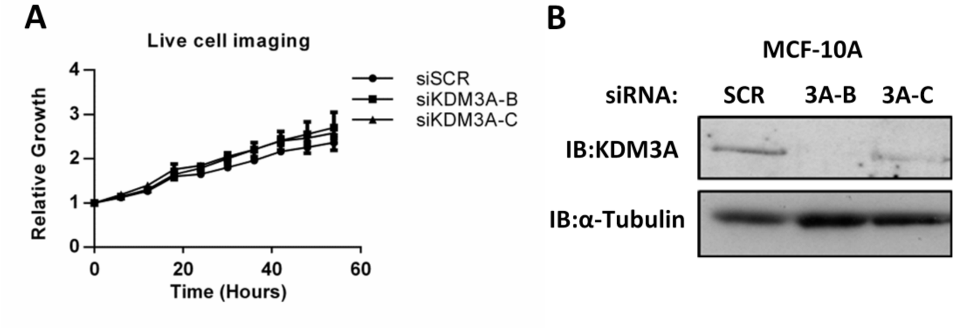

Supplementary Figure S8

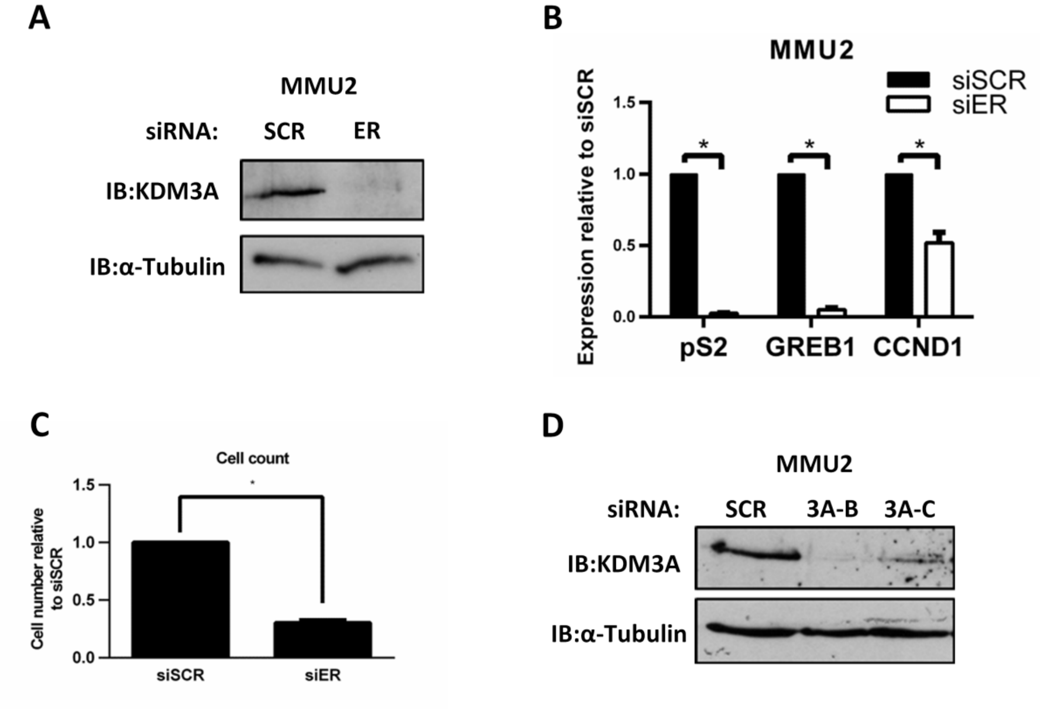

Supplementary Figure S9

GO Category	GO term	Number of genes	% of gene list	P-value	Fold enrichment
GOTERM_BP_FAT	cell cycle	207	13.24	9.56E-52	3.03
GOTERM_BP_FAT	M phase	128	8.19	4.97E-51	4.41
GOTERM_BP_FAT	cell cycle phase	144	9.21	1.21E-50	3.96
SP_PIR_KEYWORDS	cell cycle	146	9.34	5.18E-50	3.96
GOTERM_BP_FAT	cell cycle process	168	10.75	2.50E-48	3.37
GOTERM_BP_FAT	M phase of mitotic cell cycle	98	6.27	4.39E-44	4.95
GOTERM_BP_FAT	mitosis	97	6.21	5.36E-44	4.99
GOTERM_BP_FAT	nuclear division	97	6.21	5.36E-44	4.99
GOTERM_BP_FAT	mitotic cell cycle	127	8.13	1.30E-43	3.89
GOTERM_BP_FAT	organelle fission	97	6.21	3.89E-42	4.79
SP_PIR_KEYWORDS	mitosis	83	5.31	1.31E-41	5.65
SP_PIR_KEYWORDS	cell division	97	6.21	3.26E-39	4.59
GOTERM_BP_FAT	DNA metabolic process	142	9.09	2.65E-38	3.23
GOTERM_BP_FAT	DNA replication	79	5.05	2.91E-34	4.79
GOTERM_BP_FAT	cell division	98	6.27	4.51E-32	3.75
SP_PIR_KEYWORDS	dna replication	46	2.94	2.51E-26	6.48
GOTERM_BP_FAT	chromosome segregation	44	2.82	2.10E-24	6.09
KEGG_PATHWAY	DNA replication	27	1.73	9.57E-20	7.99
KEGG_PATHWAY	DNA replication	27	1.73	9.57E-20	7.99
GOTERM_BP_FAT	DNA repair	77	4.93	1.35E-19	3.09

Supplementary Table S1: GO enrichment analysis of identified gene clusters – top 20 gene clusters ordered by P-value

**siRNA Name**	**Sequence (5'-3')**
siKDM3A-B	CAAACUGCCCUUGUUCAAA
siKDM3A-C	GAGAUACUGCUUGGCUGUA
siER	GGCAUGGAGCAUCUCUACA

Supplementary Table S2: siRNA sequences

Antibody	Use<br>Western Analysis	 Immunoprecipitation	 Immunofluorescence
KDM3A A301-539A - Bethyl laboratories	x		
KDM3A ab80598 - Abcam		x	
ER (D-12) sc-805 - Santa Cruz Biotechnology	x	x	
α-tubilin T9026 - Sigma	x		
Histone H3 ab1791 - Abcam	x		
H3K9me1 C15410065 - Diagenode		x	x
H3K9me2 C15410060 - Diagenode	x	x	x
H3K9me3 C15410056 - Diagenode			x
V5 (C-9) sc271944 - Santa Cruz Biotechnology			x
**Isotype Controls**			
HA (F-7) sc7392 Santa Cruz Biotechnology		x	
Rabbit IgG - Diagenode		x	
Secondary Antibody			
Polyclonal Swine Anti-Rabbit HRP - DAKO	x		
Polyclonal Rabbit Anti-Mouse HRP - DAKO	x		
Alexa Fluor 594 Goat Anti-Rabbit - A11037 - Life Tech			x
Alexa Fluor 488 Goat Anti-Mouse - A11029 - Life Tech			x

Supplementary Table S3: Antibody Details

mRNA Primers	Sequence 5'-3'
RPL13A F	CCTGGAGGAGAAGAGGAAAGAGA
RPL13A R	TTGAGGACCTCTGTGTATTTGTCAA
KDM3A F	GGAGCTCCACATCAGGTTCATAA
KDM3A R	TTCAGCCACTTTGATGCAGC
pS2 F	GTGTCACGCCCTCCCAGT
pS2 R	GGACCCCACGAACGGTG
GREB1 F	CAAAGAATAACCTGTTGGCCCTGC
GREB1 R	GACATGCCTGCGCTCTCATACTTA
CCND1 F	ACTACCGCCTCACACGCTTC
CCND1 R	AGTCCGGGTCACACTTGATCA
PIM1 F	TATAGCCCTCCAGAGTGGAT
PIM1 R	CTGACATTCTGAAGAGACCC
CDK1 F	CCTAGCATCCCATGTCAAAAACTTGG
CDK1 R	TGATTCAGTGCCATTTTGCCAGA
CDK2 F	GCTAGCAGACTTTGGACTAGCCAG
CDK2 R	AGCTCGGTACCACAGGGTCA
CDK4 F	ATGTTGTCCGGCTGATGGA
CDK4 R	CACCAGGGTTACCTTGATCTCC
CCNA2 F	GAAGACGAGACGGGTTGCA
CCNA2 R	AGGAGGAACGGTGACATGCT
CDC25A F	CAAACCTTGACAACCGATGC
CDC25A R	ACACTGACCGAGTGCTGGAG

ChIP Primers	Sequence 5'-3'
pS2 ERE1 F	TTCCGGCCATCTCTCACTAT
pS2 ERE1 R	ATGGGAGTCTCCTCCAACCT
pS2 ERE2 F	CCATGGGAAAGAGGGACTTT
pS2 ERE2 R	TGGTCAAGCTACATGGAAGG
pS2 Control F	AATACCTGAGGACCCCAACC
pS2 Control R	TCTTCACTCTCCTCGCATTG
GREB1 ERE F	AGCAGTGAAAAAAAGTGTGGCAACTGGG
GREB1 ERE R	CGACCCACAGAAATGAAAAGGCAGCAAACT
CCND1 Enh F	CAGTTTGTCTTCCCGGGTTA
CCND1 Enh R	TCATCCAGAGCAAACAGCAG
MYC Enh F	GGCTCACCCTTGCTGATGCT
MYC Enh R	GCTCTGGGCACACATTGG
XBP1 Enh F	ATACTTGGCAGCCTGTGACC
XBP1 Enh R	GGTCCACAAAGCAGGAAAA

Supplementary Table S4: Primer Sequences
